# Pharmacological Evaluation of Antipyretic and Antioxidant Activities of 80% Methanol Root Extract and Derived Solvent Fraction of *Echinops kebericho* M. (Asteraceae) in Mice Model

**DOI:** 10.1155/2021/6670984

**Published:** 2021-03-15

**Authors:** Tesfaye Yimer, Yohannes Kelifa Emiru, Zemene Demelash Kifle, Amien Ewunetei, Meaza Adugna, Eshetie Melese Birru

**Affiliations:** ^1^Department of Pharmacy, College of Health Science, Debre Tabor University, Debre Tabor, Ethiopia; ^2^Department of Pharmacognosy, School of Pharmacy, College of Medicine and Health Sciences, University of Gondar, Gondar, Ethiopia; ^3^Department of Pharmacology, School of Pharmacy, College of Medicine and Health Sciences, University of Gondar, Gondar, Ethiopia

## Abstract

**Background:**

Toxicity and untoward effects are very ostensible in most standard drugs including antipyretic agents. Searching for conceivable antipyretic drugs with minimal toxicities and side effects from traditional plants is a growing concern to date. *Echinops kebericho* M. (Asteraceae) is one of the most prominent traditional medicinal plants, which is frequently testified for its traditionally claimed uses of treating fever and different infectious and noninfectious disorders by traditional healers in Ethiopian folk medicine. However, this plant has not been scientifically assessed for its traditionally claimed uses. This study therefore is aimed at investigating the antipyretic and antioxidant activities of 80% methanol root extract and the derived solvent fraction of *Echinops kebericho* M. in mouse models.

**Methods:**

Successive solvent maceration with increased polarity was used as the method of extractions, and chloroform, ethyl acetate, methanol, and water were used as solvents. After extraction, the crude extract and its derived solvent fractions were assessed for their antipyretic activities using yeast-induced pyrexia while, the antioxidant activities were measured in vitro using the diphenyl-2-picrylhydrazyl (DPPH) assay method. Both the extract and solvent fractions were evaluated at the doses of 100, 200, and 400 mg/kg for its antipyretic activities, and the antioxidant activity was evaluated at the doses of 50, 100, 200, 400, 600, 800, and 1000 mg/kg. The positive control group was treated with standard drug (ASA 100 mg/kg), while normal saline-receiving groups were assigned as negative control.

**Result:**

*E*. *kebericho* crude extract along with its derived solvent fractions showed statistically significant (*p* < 0.05, 0.01, and 0.001) temperature reduction activities. The maximum percentage of temperature reduction was observed by the highest dose (400 mg/kg) of the crude extract. The aqueous fraction also showed significantly (*p* < 0.05 and 0.01) higher temperature reduction than those of ethyl acetate and chloroform fractions. The free radical scavenging activities of the crude extract were also significantly high at the maximum dose, and the aqueous fraction showed the significantly highest antioxidant activity.

**Conclusion:**

In general, the data obtained from the present study clarified that the extract possessed significant antipyretic and antioxidant activities, upholding the traditionally claimed use of the plant.

## 1. Introduction

Fever is a common medical sign once the human's body temperature goes above the normal range (36.5–37.5°C) secondary to infection, tissue damage, inflammation, malignancy, graft rejection, and other inflammatory disease conditions [1, 2]. It is the body's natural defense to create an environment where an infectious agent or damaged tissue cannot survive since many of the microbial agents that cause infection grow supreme at normal body temperatures and their growth is inhibited by temperatures in the fever range [2]. Thus, a lack of fever may contribute to lower resistance to infection, delayed recovery, and suboptimal outcome. Lower febrile responses to infection are associated with a higher mortality rate and poor prognosis of any human disorders [3].

Handling of human illnesses with traditional medicinal plants has been an integral part of traditional medicine for centuries nationwide. Significantly, herbal medicines play a valuable role in developed, as well as developing, countries in improving primary healthcare for the reason that they have effective biological and medicinal properties with easy accessibility and low costs [4]. Ethiopia is endowed with leftovers of traditional medicinal plants with wide diversity of active secondary phytoconstituents that are used for treating a variety of human ailments including fever, inflammation, and oxidative stress [5, 6]. *Echinops kebericho* M. is among the widely used traditional medicinal plants in Ethiopian folk medicine which is frequently testified for its potential on scavenging free radicals and antipyretic potentials by traditional medicine practitioners in different parts of Ethiopia [7].

Conventionally, *E. kebericho* has been used for the relief of various infectious and noninfectious diseases including fever, headache, cough, diarrhea, malaria, stomach ache, and typhus and also used as taenicides by traditional healers in numerous preparations [8]. For instance, the infusion and inhalation (after burning) of the root is used to heal cough and head ache, respectively, whereas inhalation of the leaf and steam after burning is used to be relieved from inflammation which is habitually known as “mich” by old-style therapists [9]. It is also stated that the *Echinops* species is used by traditional healers for the treatment of a variety of medical conditions in different preparations. The dried and/or fresh root is fumigated for the prevention of devil sickness, and also, the dried and/or fresh root is crushed and mixed with water, and then after, a cup of the mixture is taken orally to treat head ache. Moreover, fumigation of dried and/or fresh root is used for treating malaria [10]. The dried root of *E*. *kebericho* is also mixed with coffee and taken orally to be relieved from toothache, head ache, and vomiting, by a traditional healer [11].

Traditional healers also use *E*. *kebericho*, by inhaling the dried root to heal inflammation and the evil eye [12]. Inhalation, infusion and smoking of the bulbs of *E. kebericho* are reported to be used for treating cough and head ache conventionally [13].

Despite of the frequently conveyed evidences about the traditionally claimed uses of *E*. *kebericho* by traditional healers, not any scientific reports regarding its antipyretic and antioxidant activities have been found in literatures so far. So, it deemed judicious to investigate the antioxidant and antipyretic activities of the plant scientifically upholding its traditionally claimed uses. The present study is aimed at investigating the antipyretic and antioxidant activities of 80% methanol root extract and derived solvent fraction of *E. kebericho* M. in mouse models.

## 2. Materials and Methods

### 2.1. Materials and Instruments

A rotary evaporator (Yamato, Japan), lyophilizer (Operon, OPR-FDU-5012, Korea), electronic balance (KERN-ALJ 220-4, Germany), tissue drying oven (MEDITE Medizintechnik, Germany), syringes with needles, and feeding tube were used with their respective function.

### 2.2. Drugs and Chemicals

Normal saline (H. R., Leuven, Belgium), distilled water (Ethiopian Pharmaceutical Manufacturing Factory, Ethiopia), absolute methanol (Indenta Chemicals, India), brewer's yeast (Titan Biotech Ltd., India), and aspirin, obtained from the respective vendors, were used in the experiment.

### 2.3. Collection, Identification, and Preparation of Plant Materials

The roots of *E. kebericho* were collected from around Debre Tabor town in South Gondar Zone of the Amhara regional state which is located in the northwest direction and 667 km away from the capital city Addis Ababa. The plant was then authenticated by botanists in the Department of Biology, College of Natural and Computational Sciences, University of Gondar, where a specimen with voucher number 002TYT/2019 was deposited for further reference.

### 2.4. Preparation of the Extract

After collecting the experimental plant from its indigenous place, the roots were separated and washed with tap water to remove dusts and any other debris present on it. The roots were then air dried under a shaded area at room temperature and pulverized using a mortar and pestle to get a coarse powder. A total of 3.50 kg powdered root was macerated using 80% methanol. The contents were shaken manually each day and allowed to remain within the solvent for 3 days. After 3 days, the extract was filtered first using gauze and then by a Whatman filter paper (no. 1). The marc was remacerated twice using the same volume of solvent to exhaustively extract the plant material. After extraction was completed, the solvent was evaporated under a vacuum using a rotary vapor and oven at 40°C. The resulting solution was then placed in a deep freezer operating at −20°C till it forms solid ice, and then, the remaining solvent (water) was removed using a lyophilizer. After everything, a brownish gummy residue of the crude extract weighing 224 mg was obtained, giving rise to a percentage yield of 14.93%. Then, the resulting crude extract was kept a within deep refrigerator (−20°C) till the commencement of the next procedure.

### 2.5. Preparation of the Solvent Fraction

The dried crude extract was further fractionated using sequential solvent partitioning by different solvents of increasing polarity (chloroform, ethyl acetate, and distilled water) to get different solvent fractions. Eighty grams of the extract was suspended in 400 ml of distilled water in a separatory funnel. The aqueous portion was partitioned three times with 400 ml of chloroform to obtain chloroform fraction. Then, aqueous residue was further fractionated three times with 400 ml of ethyl acetate to obtain the ethyl acetate fraction. Finally, the aqueous solution was collected as the third fraction. The chloroform and ethyl acetate fractions were concentrated in a hot air oven under 40°C. The aqueous fraction was frozen in a refrigerator overnight and then dried using a lyophilizer. The % yield of the dried fractions was calculated and the fractions obtained were put in airtight bottles and stored in a refrigerator at −4°C until being used.

### 2.6. Experimental Animals

Healthy adult Swiss albino mice of either sex (25–35 g and 6–8 weeks of age) were purchased from the Ethiopian Health and Nutrition Research Institute (EHNRI). All mice were fed with commercial pellets and have had access to water ad libitum. The mice were acclimatized for a week before commencement of the experiment in all procedures to minimize stress. All mice used in this study were handled in accordance with the internationally accepted standard guidelines for use of laboratory animals [14].

### 2.7. Animal Grouping and Dosing

Swiss albino mice of either sex (25–35 g) were randomly divided into twelve groups of six mice each. Group I was assigned as negative control and received vehicles. Group II was served as positive control and treated with standard drugs. Groups III–XII were used as test groups and given the extract of 100, 200, and 400 mg/kg and aqueous, ethyl acetate, and chloroform fractions of similar doses with the crude, respectively. Doses were selected based on the acute toxicity study done previously [7, 15]. All treatment administrations were performed orally and the maximum volume administered was 0.015 ml/kg.

## 3. Evaluation of Antipyretic Activity

### 3.1. Brewer's Yeast-Induced Pyrexia

Yeast-induced fever (pathogenic fever) which is the most common model for investigating the antipyretic potentials of natural products and synthetic substances was used to detect the antipyretic potential of *EK* extract [16, 17]. Swiss albino mice of either sex were divided into twelve groups (*n* = 6) and fasted over night with free water access. The initial basal rectal temperature of each mouse was measured using a digital thermometer by inserting a thermistor probe about 3 cm into the rectum. Fever (pyrexia) was induced in all mice by injecting 30% *w*/*v* yeast extract powder suspension in 0.9% normal saline (10 ml/kg (3 g/kg)) below the nape of the neck subcutaneously. The rectal temperature of each mouse was again recorded after 18 hrs of yeast administration to attune with the stable or plateau phase of fever, i.e., an appropriate time to test antipyretic activity of *EK* extract. Only mice showing an increase in temperature of at least 0.5°C after yeast injection were used for the experiment. After everything, each group of the mice was treated with standard drug: group I (100 mg/kg ASA), vehicle group II (10 ml/kg normal saline), and different doses of the extract, i.e., group III (100 mg/kg), group IV (200 mg/kg), and group V (400 mg/kg). All administrations were performed orally using oral gavage. Finally, the temperature of each mouse was measured at 0.5, 1, 1.5, 2, 2.5, and 3 hours after dosing. Percentage reduction in rectal temperature was calculated considering the total fall in temperature to the normal level as described in the following [18, 19]:

(1) $$\begin{array}{c} \%\text{inhibition}=\frac{\text{yeast}‐\text{induced pyrexia}-\text{posttreatment }T}{\text{yeast}‐\text{induced pyrexia}}*100. \end{array}$$

### 3.2. Evaluation of Antioxidant Activity

Several concentrations ranging from 50 to 1000 *μ*g/ml of the crude and solvent fractions of *E. kebericho* were tested for antioxidant activity in the DPPH model. Ascorbic acid was used as the standard drug for the determination of the antioxidant activity by the DPPH method [20, 21].

### 3.3. Statistical Analysis

Analysis of results was done using Statistical Package for Social Sciences (SPSS) software version 21. All results obtained were expressed as mean ± standard error of mean (SEM) of responses. The statistical significance was determined using one-way analysis of variance (ANOVA) followed by the Tukey post hoc test to compare variations among groups, and the results were considered significant at *p* < 0.05. The analyzed data were then presented using tables and graphs where necessary.

## 4. Results

### 4.1. Antipyretic Activity

#### 4.1.1. Brewer's Yeast-Induced Pyrexia in Mice

The mean temperature reductions produced by all test doses of the crude methanolic extract, aqueous, ethyl acetate, and chloroform fractions of *E*. *kebericho* were significant as compared to that of the negative control (Table 1). The percentage temperature reductions of the crude methanolic extract of *EK* at all test doses employed (100, 200, and 400 mg/kg) and ASA (100 mg/k) were 4.12%, 6.5%, 6.95%, and 7.5%, respectively, as compared to vehicle-receiving group (Table 2). The mean *T* reduction potential of the crude extract was found to increase in a dose-dependent manner. The fever reduction effect was statistically significant in 200 and 400 mg/kg (*p* < 0.01 and *p* < 0.001) doses of the crude extract starting at 1 hr after administration, and the effect persisted till the 3^rd^ time of observation (*p* < 0.001), while the effect of the lower dose of the crude extract (100 mg/kg) was observed to be significant (*p* < 0.05) from 1.5 hr after administration onwards (*p* < 0.01).The antipyretic effect of the crude extract at its maximum dose level (400 mg/kg) was commensurable to that of the reference drug (ASA 150 mg/kg) (Table 1).

The maximum antipyretic effect of AF (%*T* reduction) at its test doses employed (100, 200, and 400 mg/kg) was observed at the 3^rd^ time of observation with the respective percentage values of 3.9%, 5.1%, and 6.9% (Table 2). This percentage of *T* reduction effect was found to be increasing in a dose-dependent manner. Temperature reduction effects of the middle and high doses of AF fraction of *EK* (200 and 400 mg/kg) were statistically significant (*p* < 0.01 and *p* < 0.001) starting from 1 hr of administration, while the effect of the lower dose (100 mg/kg) was statistically significant (*p* < 0.05) from 1.5 hrs of administration onwards (Table 1).

On the other hand, the antipyretic effects of EAF of *EK* at its test doses (100, 200, and 400 mg) were statistically significant starting from the 1^st^ observation (*p* < 0.05 and *p* < 0.01) and *T* reduction activities of all doses persisted till the last observation. Each EAF of *EK* (100, 200, and 400 mg/kg) produced antipyretic percentages of 0.92%, 2.45%, and 2.54%, respectively, at the 3^rd^ time of observation.

Like the ethyl acetate fraction, the chloroform fraction at its test doses (100, 200, and 400 mg/kg) started reducing yeast-induced pyrexia starting from the 1^st^ observation onwards. The antipyretic effect of the maximum dose of the ethyl acetate fraction (400 mg/kg) was significant (*p* < 0.05) starting from 1.5 hrs of observation, and the effect continued till the 3^rd^ time of observation (*p* < 0.01) (Table 1).

#### 4.1.2. Antioxidant Activity

**Figure 1:**
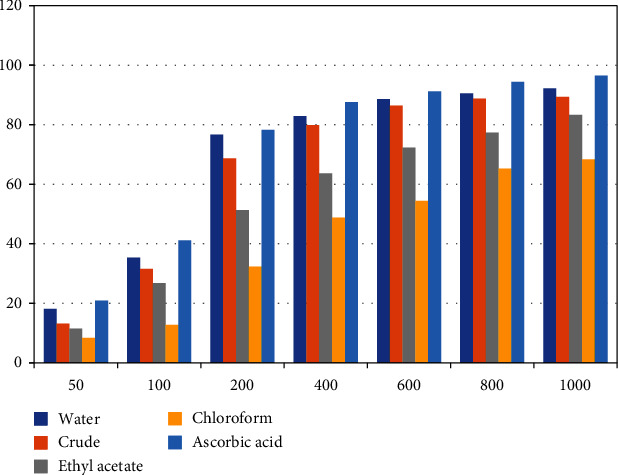
DPPH free radical scavenging activity of crude and solvent fractions of *Echinops kebericho.*

Several concentrations ranging from 50 to 1000 *μ*g/ml of the crude and solvent fractions of *EK* were tested for antioxidant activity in the DPPH model. The aqueous fraction exhibited a maximum antioxidant activity (92.19%) with an IC_50_ value of 4.11 *μ*g/ml (Table 3). There was a dose-dependent increase in the percentage antioxidant activity for all concentrations tested. Ascorbic acid was used as the standard drug for the determination of the antioxidant activity by the DPPH method. Ascorbic acid at a concentration of 1000 *μ*g/ml exhibited a percentage inhibition of 96.49% and with an IC_50_ value of 2.76 *μ*g/ml (Figure 1). The IC_50_ value was observed to be 5.89 *μ*g/ml for the crude extract, 9.58 *μ*g/ml for the ethyl acetate fraction, and 15.00 *μ*g/ml for the chloroform fraction (Figure 1).

## 5. Discussion

In this study, the potential antipyretic and antioxidant effects of hydromethanol crude extract and solvent fractions of *EK* were investigated using different animal models. In brewer's yeast-induced pyrexia, the crude extract, aqueous, and ethyl acetate fractions showed significant (*p* < 0.01 and *p* < 0.001) temperature reduction potential than the chloroform fraction. In addition, the aqueous fraction exhibited a maximum antioxidant activity in the DPPH model than those in crude extract, ethyl acetate, and chloroform fractions.

In the evaluation of antipyretic activities of hydromethanol root extract of *EK* and its solvent fraction, yeast-induced pyrexia, also called pathogenic fever, was employed. Induction of 30% *w*/*v* freshly prepared yeast in 0.9% normal saline subcutaneously below the nape of the neck of the mouse-induced fever (0.01 mg/g) after 18 hrs of induction [19]. The presence of proteins in yeast is linked to fever via a variety of inflammatory reactions in this method. The production of proinflammatory cytokines such as IL-1*β* and IL-6, IFN-*α* and TNF-*α*, and PGs like PGE_2_ and PGI_2_ is responsible for elevating the body temperature by acting on the brain which sets the thermoregulatory center at a lower-temperature regulatory area of the hypothalamus [22].

In the present study, 80% methanolic root extract of *EK* at the three test doses employed (100, 200, and 400 mg/kg) demonstrated a significant fever reduction effect (*p* < 0.05, *p* < 0.01, and *p* < 0.001, respectively) after 1 hr of administration and the effects persisted significantly at the 3^rd^ time of observation in all test doses (*p* < 0.05, *p* < 0.01, and *p* < 0.001, respectively) as compared with the negative control. The maximum fever reduction effect in all test doses of the extract (100, 200, and 400 mg/kg) was observed at the 3^rd^ time of observation with the respective percentage values of 4.12%, 6.50%, and 6.95% (Table 2). All the values gained were as per mean temperature change from the pre-extract/drug/vehicle value over each hr period and predrug yeast-induced temperature recorded for the same mice. The effects of temperature reduction potentials of the extract were found to be significant in a dose-dependent manner, i.e., the lower dose of the extract (100 mg/kg) showed significantly lower effect than the middle dose (200 mg/kg) (*p* < 0.01) and high dose (400 mg/kg) (*p* < 0.001) while the middle dose showed significantly lower antipyretic activity (*p* < 0.05) than the higher dose and the higher dose of the extract showed comparable temperature reduction potential with the standard drug (*p* > 0.05) (ASA 100 mg/kg) from the 1^st^ hr of observation and the effect continued throughout the observation times with the respective values of 6.95% and 7.50% (Figure 1).

The aqueous fraction (AF), at its lower and middle doses employed (100 mg/kg and 200 mg/kg), showed statistically significant *T* reduction effect in a dose-dependent manner starting from the 1^st^ hour of observation onwards while the maximum dose (400 mg/kg) showed statistically significant antipyretic effect starting from 0.5 hrs of observation onwards (Table 1). The aqueous fraction showed the highest antipyretic effect, suggesting that the active phytoconstituents of the extract responsible for temperature reduction might be majorly polar and dissolved with more polar solvents like distilled water.

On the other hand, EAF showed significantly lower the antipyretic activity than the aqueous fraction (*p* < 0.05) and significantly higher antipyretic activity than the chloroform fraction (*p* < 0.01). The time-dependent antipyretic effects of the crude extract and the solvent fraction suggested that the effects also have a potential kinetic profile (Table 1).

The antipyretic activity of the crude extract and its different solvent fractions shown from this study is in line with previous reports which stated that plants having analgesic and anti-inflammatory potentials may possibly possess a significant antipyretic activity [19, 23].

The presence of the active chemical constituents such as steroids, terpenoids alkaloids, and saponins in the root extract of *EK* [24] played a significant role in the antipyretic activity of the extract. Steroids and terpenoids have exerted their antipyretic effect through inhibiting the activity of prostaglandin synthetase, the enzyme that stimulates the production and release of PGs, the primary mediator in fever induction, while flavonoids inhibit elevating temperature by suppressing mediators like PGs responsible for fever, through its action against the release of AA or by interfering with the eicosanoid biosynthesis pathways involved in fever production [16, 22].

Since the lowering of temperature was almost in a similar manner to that of the reference drug, ASA, the proposed antipyretic property of the extract can be assumed to be through the interference of PG synthesis and inhibition of cytokine release which play a major role for the elevation of body temperature [18, 22].

## 6. Conclusion and Recommendation

Standing from the results gained from the present study, it can be concluded that the experimental plant extract and its solvent fraction possessed antipyretic and antioxidant activities. The crude extract and aqueous fraction in the doses of 100, 200, and 400 mg/kg significantly reduce the temperature of pyretic mice as revealed from the observation that the average percentage of antipyretic activity increased with the concentration of the extracts and its solvent fractions (400 mg/kg) as compared with the negative control groups. It is presumed that the presence of active phytoconstituents including flavonoids may be contributed to the antipyretic activities of both the extract and its solvent fraction through inhibiting the synthesis and release of different pyrogens including PGs. The current findings put scientific evidence about the traditionally claimed uses of *E. kebericho* for high fever conditions in Ethiopian folk medicines.

Further constituent isolation, binding studies, and electrophysiological procedures may be useful to fully elucidate the antipyretic and antioxidant effects and specific mechanisms of *E. Kebericho.*

## Figures and Tables

**Table 1:** *T* reduction potential of crude, aqueous, ethyl acetate, and chloroform fractions of *EK*.

Groups	Baseline *T* (°C)	*T* 18 hr after yeast(°C)	*T* after treatment (°C, mean ± SEM)					
			+0.5 hr	+1 hr	+1.5 hr	+2 hr	+2.5 hr	+3 hr
ASA 100 mg/kg	37.02	38.45 ± 0.16	37.87 ± 0.14^b3c3^	37.13 ± 0.19^b1c1d2e3^	36.53 ± 0.16^b1c1d1e1^	36.10 ± 0.07^b1c1d1e3^	35.90 ± 0.07^b3c3d1e1^	35.57 ± 0.09^b3c3^
NS 10 ml/kg	37.1	38.27 ± 0.08	38.65 ± 0.15	38.88 ± 0.14	38.95 ± 0.13	39.13 ± 0.06	39.38 ± 0.07	39.48 ± 0.06
*EK* 100 mg/kg	37.18	38.83 ± 0.17	38.62 ± 0.13	38.32 ± 0.20^a2d1e1^	38.00 ± 0.18^a3b1d2e2^	37.78 ± 0.16^a3b3d1e3^	37.53 ± 0.13^a3b3e3^	37.23 ± 0.15^a3b3e3^
*EK* 200 mg/kg	36.15	38.18 ± 0.20	37.92 ± 0.27	37.38 ± 0.28^b3c1^	36.95 ± 0.28^b3c2^	36.55 ± 0.33^b3c1^	36.12 ± 0.36^b3c3^	35.70 ± 0.36^b3^
*EK* 400 mg/kg	36.75	38.35 ± 0.19	37.90 ± 0.20	37.35 ± 0.17^b3c1^	36.93 ± 0.13^b3c2^	36.43 ± 0.14^b3c3^	36.07 ± 0.12^b3c3^	35.68 ± 0.06^b3c3^
AQ 100 mg/kg	36.8	38.83 ± 0.17	38.61 ± 0.13	38.31 ± 0.20^a2d3e3^	38.00 ± 0.18^a1d3e3^	37.78 ± 0.15^a1b1d2e3^	37.53 ± 0.12^a1b1d1e1^	37.23 ± 0.14^a1b1d1e3^
AQ 200 mg/kg	36.9	38.18 ± 0.20	37.91 ± 0.27	37.38 ± 0.27^b1c3^	36.95 ± 0.15^b1c3^	36.55 ± 0.33^b1c2^	36.11 ± 0.37^b1c1^	35.70 ± 0.35^b1c1^
AQ 400 mg/kg	36.9	38.35 ± 0.19	37.90 ± 0.20^b^	37.35 ± 0.16^b1c3^	39.93 ± 0.08^b1c3^	36.43 ± 0.14^b1c2^	36.06 ± 0.12^b1c1^	35.68 ± 0.06^b1c3^
EA 100 mg/kg	36.8	38.3 ± 0.17	38.13 ± 0.48^b3^	38.18 ± 0.14^a2d2^	37.9 ± 0.13^a1b1^	37.91 ± 0.06^a1b1d3e3^	37.93 ± 0.06^a1b1d2e3^	37.84 ± 0.06^a1b1c2e1^
EA 200 mg/kg	36.9	38.12 ± 0.20	37.93 ± 0.12^b2^	37.81 ± 0.16^b1^	37.64 ± 0.15^a1b1^	37.53 ± 0.13^a1b1c3^	37.31 ± 0.14^a1b1c3^	37.28 ± 0.12^a1b1c2^
EA 400 mg/kg	36.9	38.03 ± 0.19	37.85 ± 0.07^b1^	37.75 ± 0.12^b1^	37.56 ± 0.08^a1b1^	37.54 ± 0.12^a1b1c3^	37.32 ± 0.10^a1b1c1^	37.02 ± 0.12^a1b1c1^
CF 100 mg/kg	36.8	38.3 ± 0.17	38.65 ± 0.48^a2e3^	38.88 ± 0.14^a1d3e2^	38.9 ± 0.13^a1d1e1^	39.1 ± 0.06^a1d1e1^	39.3 ± 0.06^a1d1e1^	39.4 ± 0.06^a1d1e3^
CH 200 mg/kg	36.9	38.12 ± 0.20	38.13 ± 0.12	38.08 ± 0.16^a2b3c3^	37.9 ± 0.15^a1b1c2^	37.9 ± 0.13^a1b1c1^	37.9 ± 0.14^a1b1c1^	37.8 ± 0.12^a1b3b1^
CH 400 mg/kg	36.9	38.03 ± 0.19	37.95 ± 0.12^b3c3^	37.95 ± 0.12^a3b2c2^	37.6 ± 0.08^a1b1c1^	37.5 ± 0.12^a1b1c1d3^	37.3 ± 0.10^a1b1c1^	37.2 ± 0.12^a1b1c3^

Analysis was performed with one-way ANOVA followed by the Tukey post hoc test. Data was expressed in mean ± SEM. *n* = 6. ASA: acetyl salicylic acid (100 ml/kg); NS: normal saline (10 ml/kg); *EK* 100 mg/kg: *Echinops kebericho* extract (100 mg/kg); *EK* 200 mg/kg: *Echinops kebericho* extract (200 mg/kg); *EK* 400 mg/kg: *Echinops kebericho* extract (400 mg/kg); AQ 100 mg/kg: aqueous fraction (100 mg/kg); AQ 200 mg/kg: aqueous fraction (200 mg/kg); AQ 400 mg/kg: aqueous fraction (400 mg/kg); EA 100 mg/kg: ethyl acetate fraction (100 mg/kg); EA 200 mg/kg: ethyl acetate fraction (200 mg/kg); EA 400 mg/kg: ethyl acetate fraction (400 mg/kg); CH 100 mg/kg: chloroform fraction (100 mg/kg); CH 200 mg/kg: chloroform fraction (200 mg/kg); CH 400 mg/kg: chloroform fraction (400 mg/kg). ^a^As compared to +ve control; ^b^as compared to −ve control; ^c^as compared to 100 mg/kg *EK*; ^d^as compared to 200 mg/kg *EK*; ^e^as compared to 400 mg/kg *EK*; ^3^*p* < 0.001; ^2^*p* < 0.01; ^1^*p* < 0.

**Table 2:** Percent temperature reduction potentials of crude extract, aqueous, ethyl acetate, and chloroform fractions of *E. kebericho.*

Groups	+0.5 hr	+1 hr	+1.5 hr	+2 hr	+2.5 hr	+3 hr
ASA 150 mg/kg	1.51	3.42	4.98	6.11	6.63	7.49
*EK* 100 mg/kg	0.56	1.33	2.14	2.70	3.34	4.12
*EK* 200 mg/kg	0.70	2.10	3.23	4.27	5.41	6.50
*EK* 400 mg/kg	1.17	2.61	3.69	4.10	5.95	6.95
AQ 100 mg/kg	0.35	1.04	1.86	2.34	3.20	3.90
AQ 200 mg/kg	0.61	1.92	3.19	4.11	5.07	6.38
AQ 400 mg/kg	1.17	2.56	3.69	5.08	5.95	6.91
EA 100 mg/kg	0.13	0.22	0.61	0.65	0.61	0.92
EA 200 mg/kg	0.52	0.31	1.31	1.70	2.05	2.45
EA 400 mg/kg	0.66	0.88	1.23	1.53	2.02	2.54
CH 100 mg/kg	0.09	0.22	0.61	0.65	0.61	0.92
CH 200 mg/kg	0.52	0.83	1.31	1.70	2.05	2.44
CH 400 mg/kg	0.66	0.87	1.22	1.53	2.02	2.54

Data was expressed as mean ± SEM. *n* = 6; ASA: acetyl salicylic acid (100 mg/kg); NS: normal saline (10 ml/kg); *EK* 100 mg/kg: *Echinops kebericho* extract (100 mg/kg); *EK* 200 mg/kg: *Echinops kebericho* extract (200 mg/kg); *EK* 400 mg/kg: *Echinops kebericho* extract (400 mg/kg); AQ 100 mg/kg: aqueous fraction (100 mg/kg); AQ 200 mg/kg: aqueous fraction (200 mg/kg); AQ 400 mg/kg: aqueous fraction (400 mg/kg); EA 100 mg/kg: ethyl acetate fraction (100 mg/kg); EA 200 mg/kg: ethyl acetate fraction (200 mg/kg); EA 400 mg/kg: ethyl acetate fraction (400 mg/kg); CH 100 mg/kg: chloroform fraction (100 mg/kg); CH 200 mg/kg: chloroform fraction (200 mg/kg); CH 400 mg/kg: chloroform fraction (400 mg/kg). *T*: temperature.

**Table 3:** Antioxidant activities and IC_50_ values of the crude and solvent fractions of *Echinops kebericho*.

Extract	50 *μ*g/ml	100 *μ*g/ml	200 *μ*g/ml	400 *μ*g/ml	600 *μ*g/ml	800 *μ*g/ml	1000 *μ*g/ml	IC50 (*μ*g/ml)
Crude	13.23 ± 2.01	31.56 ± 2.14	68.72 ± 1.37	79.83 ± 1.57	86.43 ± 0.87	88.82 ± 1.49	89.32 ± 0.96	5.89 ± 1.02
EA	11.57 ± 1.89	26.81 ± 3.07	51.32 ± 2.47	63.65 ± 2.22	72.28 ± 1.37	77.34 ± 1.77	83.27 ± 1.57	9.58 ± 0.68
AQ	18.12 ± 3.12	35.42 ± 2.88	76.72 ± 2.08	82.91 ± 3.47	88.61 ± 2.19	90.57 ± 0.94	92.19 ± 2.61	4.11 ± 0.94
CH	8.38 ± 0.89	12.77 ± 0.96	32.37 ± 1.37	48.76 ± 0.67	54.37 ± 3.37	65.29 ± 2.49	68.37 ± 3.21	15 ± 1.32
Ascorbic acid	20.94 ± 1.67	41.16 ± 1.08	78.28 ± 3.07	87.61 ± 1.28	91.19 ± 0.82	94.37 ± 0.79	96.49 ± 1.47	2.76 ± 0.75

## Data Availability

All data that are analyzed are available on the hand of the corresponding author.

## Abbreviations

AA: Arachidonic acid
AF: Aqueous fraction
ASA: Acetyl salicylic acid
EAF: Ethyl acetate fraction
*EK*: *Echinops kebericho* Mesfin
IC: Effective concentration
IFN: Interferon
IL: Interleukin
PGs: Prostaglandins
TNF: Tumor necrosis factor.
